# Supplementary material for: Mechanisms governing the pioneering and redistribution capabilities of the non-classical pioneer PU.1
Source: Nat Commun. 2020 Jan 21;11:402. doi: 10.1038/s41467-019-13960-2 (PMC6972792; doi:10.1038/s41467-019-13960-2)
Supplement: Supplementary file 7 — Source data [file 41467_2019_13960_MOESM7_ESM.zip › Source_Data/FigureS2/FigureS2B_MotifScanOutput/KmeansCluster7/homerResults/motif27.similar.html]

motif27

## Information for motif27

A
G
C
T
T
G
A
C
G
C
A
T
A
G
T
C
T
C
A
G
C
G
A
T
C
T
A
G
T
G
C
A
G
A
C
T
C
G
T
A
T
A
C
G
G
A
C
T
C
T
A
G
  
Reverse Opposite:  

G
A
T
C
C
G
T
A
A
G
T
C
G
C
A
T
C
T
G
A
A
C
G
T
G
A
T
C
C
G
T
A
A
G
T
C
T
C
A
G
C
G
T
A
A
C
T
G
C
T
G
A
  

|  |  |
| --- | --- |
| p-value: | 1e-49 |
| log p-value: | -1.146e+02 |
| Information Content per bp: | 1.750 |
| Number of Target Sequences with motif | 96.0 |
| Percentage of Target Sequences with motif | 2.99% |
| Number of Background Sequences with motif | 134.0 |
| Percentage of Background Sequences with motif | 0.31% |
| Average Position of motif in Targets | 106.5 +/- 51.0bp |
| Average Position of motif in Background | 122.1 +/- 65.8bp |
| Strand Bias (log2 ratio + to - strand density) | -0.6 |
| Multiplicity (# of sites on avg that occur together) | 1.02 |
| Motif File: | file (matrix) reverse opposite |

### Similar de novo motifs found

|  |  |  |  |  |  |  |  |
| --- | --- | --- | --- | --- | --- | --- | --- |
| Rank | Match Score | Redundant Motif | P-value | log P-value | % of Targets | % of Background | Motif file |
| 1 | 0.849 | A G T C A C G T G T C A A G C T G T A C C G T A A G C T T C G A C G T A T A C G C T G A | 1e-38 | -88.753454 | 2.18% | 0.20% | motif file (matrix) |
| 2 | 0.813 | A T G C G C A T T G A C T C G A A C G T C A T G T C G A C G A T C T G A T A C G | 1e-36 | -84.888896 | 6.92% | 2.45% | motif file (matrix) |
| 3 | 0.783 | A C G T A C T G C G T A A C G T C G T A A C T G A C G T | 1e-14 | -32.756891 | 3.61% | 1.54% | motif file (matrix) |
